# Ethanol exposure of human pancreatic normal ductal epithelial cells induces EMT phenotype and enhances pancreatic cancer development in KC (Pdx1‐Cre and LSL‐Kras^G12D^) mice

**DOI:** 10.1111/jcmm.17092

**Published:** 2021-12-03

**Authors:** Wei Yu, Yiming Ma, Sanjit K. Roy, Rashmi Srivastava, Sharmila Shankar, Rakesh K. Srivastava

**Affiliations:** ^1^ Kansas City VA Medical Center Kansas City Missouri USA; ^2^ Stanley S. Scott Cancer Center Louisiana State University Health Sciences Center New Orleans Louisina USA; ^3^ Department of Pharmacology Louisiana State University Health Sciences Center New Orleans Louisina USA; ^4^ Department of Genetics Louisiana State University Health Sciences Center New Orleans Louisina USA; ^5^ John W. Deming Department of Medicine Tulane University School of Medicine New Orleans Louisina USA; ^6^ Southeast Louisiana Veterans Health Care System New Orleans Louisina USA

**Keywords:** alcohol, cancer stem cells, Kras^G12D^ mice, pancreatic cancer, pluripotency, self‐renewal, transformation

## Abstract

Alcohol is a risk factor for pancreatic cancer. However, the molecular mechanism by which chronic alcohol consumption influences pancreatic cancer development is not well understood. We have recently demonstrated that chronic ethanol exposure of pancreatic normal ductal epithelial cells (HPNE) induces cellular transformation by generating cancer stem cells (CSCs). Here, we examined whether chronic ethanol treatment induces epithelial–mesenchymal transition in HPNE cells and promotes pancreatic cancer development in KC (Pdx1‐Cre, and LSL‐Kras^G12D^) mice. Our data demonstrate that chronic ethanol exposure of HPNE cells induces SATB2 gene and those cells became highly motile. Ethanol treatment of HPNE cells results in downregulation of E‐Cadherin and upregulation of N‐Cadherin, Snail, Slug, Zeb1, Nanog and BMI‐1. Suppression of SATB2 expression in ethanol‐transformed HPNE cells inhibits EMT phenotypes. KC mice fed with an ethanol‐containing diet show enhanced pancreatic cancer growth and development than those fed with a control diet. Pancreas isolated from KC mice fed with an ethanol‐containing diet show higher expression of stem cell markers (CD133, CD44, CD24), pluripotency‐maintaining factors (cMyc, KLF4, SOX‐2, and Oct‐4), N‐Cadherin, EMT‐transcription factors (Snail, Slug, and Zeb1), and lower expression of E‐cadherin than those isolated from mice fed with a control diet. Furthermore, pancreas isolated from KC mice fed with an ethanol‐containing diet show higher expression of inflammatory cytokines (TNF‐α, IL‐6, and IL‐8) and PTGS‐2 (COX‐2) gene than those isolated from mice fed with a control diet. These data suggest that chronic alcohol consumption may contribute to pancreatic cancer development by generating inflammatory signals and CSCs.

## 1 INTRODUCTION

Pancreatic cancer is the fourth leading cause of cancer‐related deaths in the US.^1^ With an overall 5‐year survival rate of 8%,^2^ pancreatic cancer has one of the poorest prognoses among all cancers.^3^ The incidence of pancreatic cancer varies significantly throughout the world, suggesting that several factors may be responsible for this deadly disease.^4^ Genetic, race, gender, environmental carcinogen, diet, and lifestyle are the primary factors for pancreatic cancer.^5^ Other factors, such as smoking, alcohol, and exposure to organochlorine or hydrocarbon solvents, have been associated with the K‐ras mutations causing pancreatic ductal adenocarcinoma (PDAC).^6^, ^7^, ^8^, ^9^ Metabolic conditions such as obesity, hypertension, dyslipidaemia, insulin resistance, and type 2 diabetes mellitus are also risk factors for pancreatic cancer.^8^, ^10^ About 5%–10% of patients with pancreatic cancer have underlying germline mutations or disorders, while the remaining percentage of cancer cases may be due to somatic mutations.^4^

Epidemiological data suggest that heavy alcohol drinking increases the risk for pancreatic cancer.^11^, ^12^, ^13^, ^14^ Alcohol intake promotes intestinal tumourigenesis and tumour invasion in genetically susceptible mice, increases in polyp‐associated mast cells, and mast cell‐mediated tumour migration in vitro,^15^ suggesting mast cell‐mediated inflammation could promote carcinogenesis.^15^ Heavy alcohol intake is associated with the risk of developing chronic pancreatitis,^16^, ^17^, ^18^ which may lead to pancreatic cancer. Alcohol drinking increases the permeability of the gut wall and translocation of lipopolysaccharide, which enhances pancreatic injury.^19^, ^20^ The effects of alcohol are modulated by polymorphisms in genes encoding enzymes for ethanol metabolism (e.g., alcohol dehydrogenases, aldehyde dehydrogenases, and cytochrome P450 2E1), folate metabolism, and DNA repair. During metabolism, ethanol is oxidized to acetaldehyde by ADH or CYP2E1.^21^, ^22^ Ethanol, acetaldehyde, and reactive oxygen species (ROS) are considered potential human carcinogens. Chronic ethanol exposure of HPNE cells induced transformation, and those transformed cells gained the phenotypes of cancer stem cells (CSCs).^23^ However, the molecular mechanism by which ethanol toxicity exerts its effects on pancreatic carcinogenesis is not well understood.

SATB2 (special AT‐rich binding protein‐2), a transcription factor and epigenetic regulator that binds DNA^24^ to regulate gene expression.^25^, ^26^, ^27^ SATB2 gene, although not expressed in healthy adults, is essential for normal mammalian development and proper facial patterning of the embryo and healthy bone development.^27^ Hyperactivation/induction of SATB2 gene causes malignant cellular transformation.^23^, ^28^, ^29^, ^30^, ^31^ SATB2 regulates transcription of pluripotency‐maintaining factors (KLF4, Oct‐4, SOX‐2, and cMyc) which form the core regulatory positive feedback‐loop for sustaining self‐renewal capacity of stem cells. It has been shown that SATB2 binds to the promoters of Bcl‐2, Bsp, Nanog, cMyc, XIAP, KLF4, and Hoxa2, suggesting a role of this gene in the regulation of cell survival, pluripotency, and proliferation.^29^ Interestingly, high levels of ROS generated by ethanol exposure can induce SATB2 expression in HPNE cells.^29^ Therefore, SATB2 protein may play a critical role in cellular transformation and carcinogenesis.

The primary goal of this paper is to examine the molecular mechanisms by which chronic ethanol exposure of HPNE cells induces EMT characteristics and ethanol feeding of KC mice enhances pancreatic cancer growth and development. To investigate the role of SATB2 at an early step of cell transformation, we utilized HPNE cells as a model to generate stem‐like cells through chronic ethanol exposure. Our data demonstrate that chronic ethanol exposure can induce EMT and oral ethanol feeding of KC mice promotes pancreatic cancer growth and development by regulating SATB2, inflammatory cytokines, PTGS‐2, stem cell markers, and pluripotency‐maintaining factors. These data suggest that excessive alcohol can promote pancreatic carcinogenesis.

## 2 MATERIALS AND METHODS

### 2.1 Cell culture conditions and reagents

Human pancreatic normal ductal epithelial (HPNE) cells were purchased from American Type Culture Collection (ATCC, Manassas, VA, USA). HPNE cells were grown in well‐defined cell culture medium as described.^32^ Foetal bovine serum (FBS), lipofectamine‐2000, and Dulbecco's Modified Eagle Medium (DMEM) were purchased from Thermo Fisher Scientific. Primers for the polymerase chain reaction (PCR) were purchased from Real Time Primers, LLC. The haematoxylin and eosin stain (H&E) was purchased from Sigma‐Aldrich, Inc.

### 2.2 Lentiviral particle production and transduction

The lentivirus production and transduction were performed as described elsewhere.^33^ In brief, lentivirus was produced by triple transfection of HEK 293T cells. Packaging 293T cells were plated in 10‐cm plates at a cell density of 5 × 10^6^ 1 day before transfection in DMEM containing 10% heat‐inactivated foetal bovine serum. A total of 293T cells were transfected with 4 µg of plasmid and 4 µg of the lentiviral vector using lipid transfection (Lipofectamine‐2000) according to the manufacturer's protocol. Viral supernatants were collected and concentrated by adding PEG‐it virus precipitation solution (System Biosciences, Palo Alto, CA) to produce virus stocks with titres of 1 × 10^8^ to 1 × 10^9^ infectious units per ml. Viral supernatant was collected for 3 days by ultracentrifugation and concentrated 100‐fold. Titres were determined on 293T cells. Cells were transduced with lentiviral particles expressing the gene of interest.

### 2.3 Quantitative real‐time PCR

Total RNA in cells was extracted by the TRIzol reagent (Invitrogen) and reverse transcribed into cDNA using High‐Capacity cDNA Reverse Transcription Kit (Thermo Fisher Scientific). qRT‐PCR was conducted using fast SYBR Green Master Mix (Thermo Fisher Scientific). The 2^−ΔΔ^ *^C^_t_* method was used to evaluate relative mRNA expressions compared with controls.

### 2.4 Motility assay

Assay for cell motility was performed as we described elsewhere.^33^, ^34^, ^35^

### 2.5 KC (Pdx1‐Cre and LSL‐Kras^G12D^) mice

KC (Pdx1‐Cre, and LSL‐Kras^G12D^) mice were generated as described elsewhere.^36^ Mice (4–6 weeks old) were fed either a control diet or ethanol‐containing liquid diet (Dyets, Inc.) as described.^37^, ^38^ Mice were fed the control diet for 1 week to acclimate to the liquid diet and then fed either the control or ethanol‐containing liquid diet (4%, v/v) for 6 months. At the end of the treatment, mice were sacrificed. Histological examination of the pancreas was performed by H&E staining as we described elsewhere,^29^ and the numbers of PanINs and PDAC were quantified.^36^

### 2.6 Statistical analysis

The mean and standard deviation (SD) were calculated for each experimental group with replicates. Differences between groups were analysed by analysis of variance (ANOVA), followed by Bonferroni's multiple comparison tests using PRISM statistical analysis software (GrafPad Software, Inc.). Significant differences among groups were calculated at *p* < 0.05.

## 3 RESULTS

### 3.1 Chronic ethanol exposure of HPNE cells induces SATB2 and EMT characteristics

**FIGURE 1:**
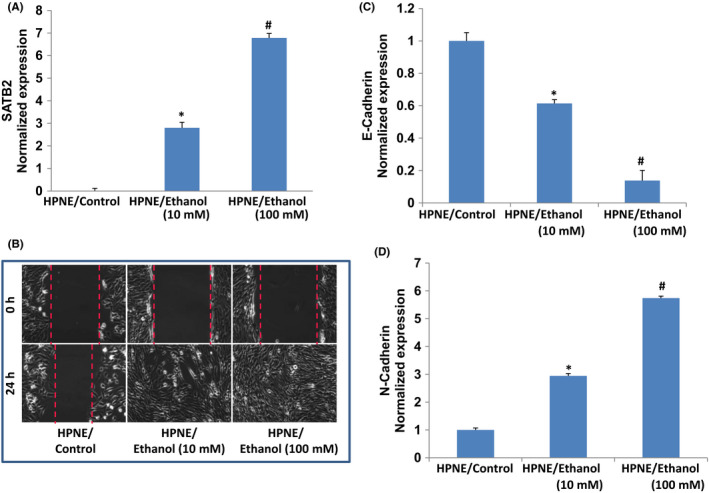
Chronic ethanol exposure of human pancreatic normal ductal epithelial (HPNE) cell induces motility and SATB2 expression. (A) Cell motility assay. HPNE and ethanol‐transformed HPNE cells (10 and 100 mM) were grown in special cell culture medium. After 12 h of cell culture, cells were scratched with the fine pipette tips. Phase contrast images of scratched cells were captured at 0 h, and after 24 h of cell growth. (B) Expression of SATB2. RNA was extracted and qRT‐PCR analysis was performed to measure the expression of SATB2 as we described elsewhere.^35^, ^41^ GAPDH was used as an internal control. Data represent mean ± SD. *, and # = significantly different from each other and HPNE/control cells, *p* < 0.05. (C and D), Expression of E‐cadherin and N‐cadherin. RNA was extracted and qRT‐PCR analysis was performed to measure the expression of E‐cadherin and N‐cadherin. GAPDH was used as an internal control. Data represent mean ± SD. *, and # = significantly different from each other and HPNE/control cells, *p* < 0.05

We have recently demonstrated that long‐term chronic exposure of HPNE cells to ethanol‐induced cellular transformation and those transformed cells in vitro gained the phenotype of cancer stem cells (CSCs).^23^ Here, we have extended those studied and further used HPNE cells as a model to assess whether chronic ethanol exposure of HPNE cells induces SATB2 expression and EMT characteristics. HPNE cells were grown in a cell culture medium in the presence or absence of ethanol (10 mM and 100 mM) for 6 months. Studies have shown the role of SATB2 in chromatin remodelling and regulation of genes, which participate in cell growth, survival, differentiation, self‐renewal, and pluripotency.^28^, ^29^, ^30^, ^31^, ^39^ We therefore examined the mechanism of ethanol‐induced EMT characteristics in HPNE cells by comparing the expression of SATB2 in HPNE control cells and ethanol‐transformed HPNE cells (HPNE/Ethanol). As shown in Figures 1A 6‐month exposure of HPNE cells to ethanol had induced the expression of SATB2 gene. As indicated before, the expression of SATB2 gene was not observed in normal HPNE cells.

Induction of EMT is one of the characteristics of transformed and cancer stem cells. The transition of a transformed cell from an epithelial–mesenchymal transition (EMT) leads to increased migratory and invasive behaviours, and thus facilitates initiation of early metastasis.^40^, ^41^ To test whether ethanol‐transformed cells gained the characteristics of EMT, we measured cell motility and the expression of E‐cadherin and N‐cadherin, which play significant roles during EMT. Both doses of ethanol (10 and 100 mM) enhanced cell motility of transformed HPNE/ethanol cells, whereas cell motility was very low in HPNE/control cells (Figure 1B). During EMT, the expression of E‐cadherin is reduced and the expression of N‐cadherin is increased.^40^, ^41^ We therefore measured the expression of E‐cadherin and N‐cadherin in ethanol‐transformed cells. Both doses of ethanol treatment inhibited the expression of E‐cadherin and upregulated the expression of N‐cadherin in HPNE/ethanol cells (Figure 1C,D). These data suggest that chronic exposure of HPNE cells to ethanol can induce EMT by regulating E‐cadherin and N‐cadherin.

### 3.2 Chronic exposure of HPNE cells to ethanol induces EMT‐transcription factors (Snail, Slug, and Zeb1), Nanog and BMI

**FIGURE 2:**
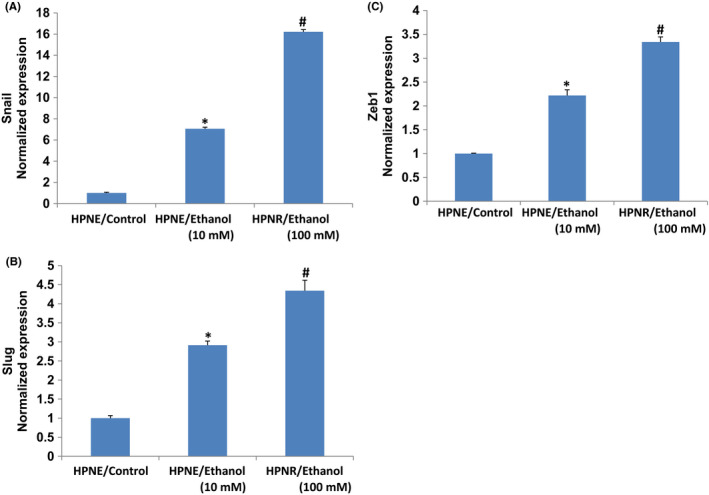
Effects of ethanol on EMT‐related transcription factor. (A–C) Expression of Snail, Slug, and Zeb1. RNA was extracted and qRT‐PCR analysis was performed to measure the expression of Snail, Slug, and Zeb1 as described elsewhere.^35^, ^41^ GAPDH was used as an internal control. Data represent mean ± SD. *, and # = significantly different from each other and HPNE/control cells, *p* < 0.05

During EMT, the expression of E‐cadherin is reduced and the expression of N‐cadherin is increased by transcription factors Snail, Slug, and Zeb1.^40^, ^41^ We therefore measured the expression of EMT‐related transcription factors. Both doses of ethanol treatment induced the expression of Snail, Slug, and Zeb1 in HPNE/ethanol cells (Figure 2). These data suggest that chronic exposure of HPNE cells to ethanol can induce EMT by regulating transcription factors Snail, Slug, and Zeb1.

**FIGURE 3:**
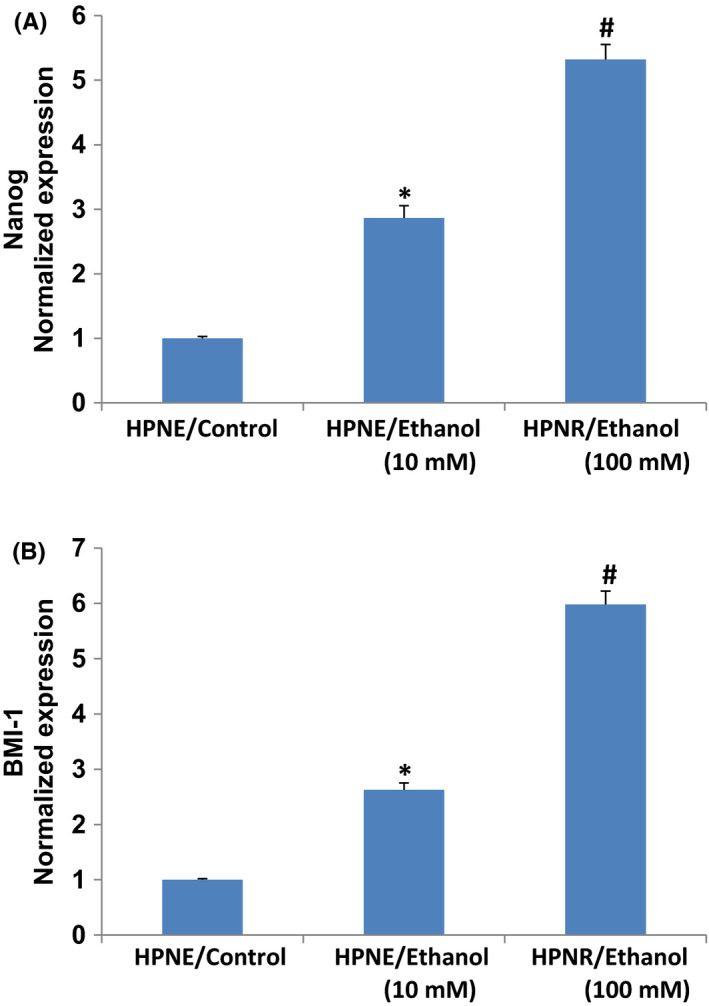
Effects of ethanol on Nanog and BMI‐1. (A–C) Expression of Nanog and BMI‐1. RNA was extracted and qRT‐PCR analysis was performed to measure the expression of Nanog and BMI‐1 by qRT‐PCR analysis. GAPDH was used as an internal control. Data represent mean ± SD. *, and # = significantly different from each other and HPNE/control cells, *p* < 0.05

NANOG is one of the key transcription factors essential for maintaining self‐renewal and pluripotency in stem cells and promotes cell migration and stemness.^33^, ^42^, ^43^ We next sought to examine the effects of ethanol on the expression of Nanog. Both doses of ethanol treatment induced the expression of Nanog in HPNE/ethanol cells (Figure 3). These data suggest that chronic exposure of HPNE cells to ethanol can regulate cell motility by modulating the expression of Nanog. These data indicate that Nanog may play an essential role in migration of ethanol‐transformed cells.

BMI‐1 has been shown to promote invasion and metastasis of pancreatic CSCs by activating PI3K/AKT singing pathway by negatively regulating PTEN.^44^ We next sought to examine the effects of ethanol on the expression of BMI‐1. Chronic ethanol (10 mM and 100 mM) exposure of HPNE cells induced the expression of BMI‐1 in HPNE/Ethanol cells (Figure 3). These data suggest that BMI‐1 may play an essential role in the migration of ethanol‐transformed cells.

### 3.3 SATB2 shRNA inhibits cell motility and regulates cadherin expression in EtOH‐transformed cells

**FIGURE 4:**
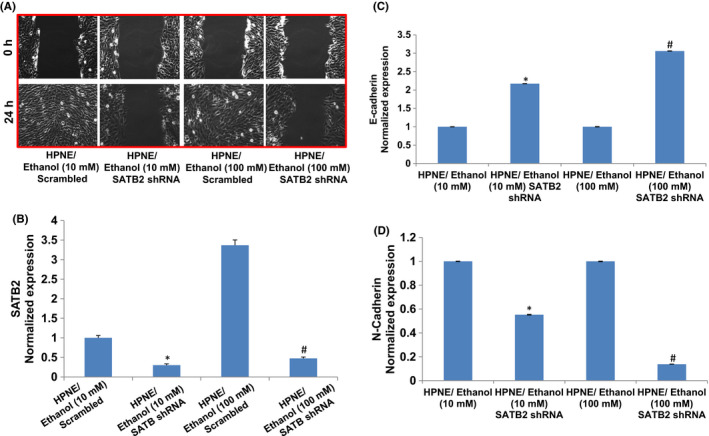
SATB2 shRNA inhibits cell motility and regulates SATB2 and cadherin expression. (A) Cell motility. Ethanol‐transformed HPNE cells (10 or 100 mM ethanol for 6 months) were transduced with either scrambled or SATB2 shRNA. Cells were grown in normal cell culture media. After 12 h of cell culture, cells were scratched with the fine pipette tips. Phase contrast images of scratched cells were captured at 0 h and 24 h of cell growth. (B) SATB2 expression. RNA was isolated, and the expression of SATB2 was measured by qRT‐PCR analysis. Data represent mean ± SD. *, or # = significantly different from scrambled control group, *p* < 0.05. (C) E‐cadherin expression. RNA was isolated, and the expression of E‐cadherin was measured by qRT‐PCR analysis. Data represent mean ± SD. *, or # = significantly different from scrambled control group, *p* < 0.05. (D), N‐cadherin expression. RNA was isolated, and the expression of N‐cadherin was measured by qRT‐PCR analysis. Data represent mean ± SD. *, or # = significantly different from scrambled control group, *p* < 0.05

To examine whether SATB2 in involved in inducing EMT, we knocked down the expression of SATB2 by shRNA in ethanol‐transformed HPNE cells, which were exposed to 10 or 100 mM ethanol for 6 months. Ethanol‐transformed HPNE cells were transduced with either scrambled or SATB2 shRNA lentiviral particles, and cell growth and colony formation were measured (Figure 4). Transduction of ethanol‐transformed HPNE cells [HPNE/Ethanol (10 mM or 100 mM)] with SATB2 shRNA viral particles inhibited the expression of SATB2 mRNA compared with that of HPNE/ethanol (10 mM or 100 mM)/scrambled cells (Figure 4A).

We next examined the effects of inhibiting SATB2 on cell motility and expression of E‐cadherins and N‐cadherins in ethanol‐transformed cells (Figure 4B–D). SATB2 shRNA inhibited cell motility in both ethanol‐transformed HPNE groups [HPNE/Ethanol (10 mM)/SATB2 shRNA and HPNE/ethanol (100 mM)/SATB2 shRNA] compared with that of scrambled groups [HPNE/ethanol (10 mM)/scrambled and HPNE/ethanol (100 mM)/scrambled]. These data suggest that SATB2 can inhibit the ability of ethanol‐transformed cells to migrate by modulating the expression of cadherins.

### 3.4 SATB2 shRNA inhibits ethanol‐induced expression of EMT‐related transcription factors in ethanol‐transformed cells

**FIGURE 5:**
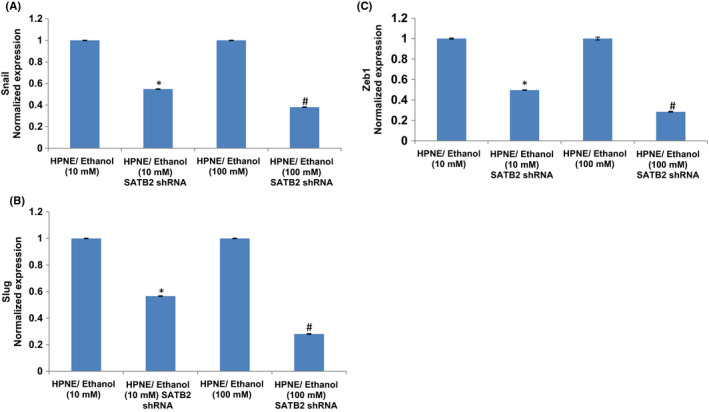
SATB2 shRNA inhibits EtOH‐induced expression of EMT‐related transcription factors in EtOH‐transformed cells. (A–C), Ethanol‐transformed HPNE cells [HPNE/ethanol (10 mM)/SATB2 shRNA, and HPNE/ethanol (100 mM)/SATB2 shRNA] were transduced with lentiviral particles expressing either scrambled or SATB2 shRNA. RNA was isolated, and the expression of Snail, Slug, and Zeb1 was measured by qRT‐PCR. Data represent mean ± SD. *, or # = significantly different from scrambled control group, *p* < 0.05

Since ethanol‐transformed HPNE cells demonstrated enhanced migratory behaviour, we next sought to examine whether inhibition of SATB2 will attenuate the expression of Snail, Slug, and Zeb1 in ethanol‐transformed cells (Figure 5A–C). SATB2 shRNA inhibited the expression of Snail, Slug, and Zeb1 in ethanol‐transformed HPNE cells [HPNE/ethanol (10 mM)/SATB2 shRNA, and HPNE/ethanol (100 mM)/SATB2 shRNA]. These data suggest that SATB2 can regulate EMT characteristics by regulating the expression of Snail, Slug, and Zeb1 in ethanol‐transformed HPNE cells (HPNE/ethanol).

### 3.5 **SATB2** **shRNA inhibits ethanol‐induced expression of Nanog and BMI‐1 in EtOH‐transformed cells**

**FIGURE 6:**
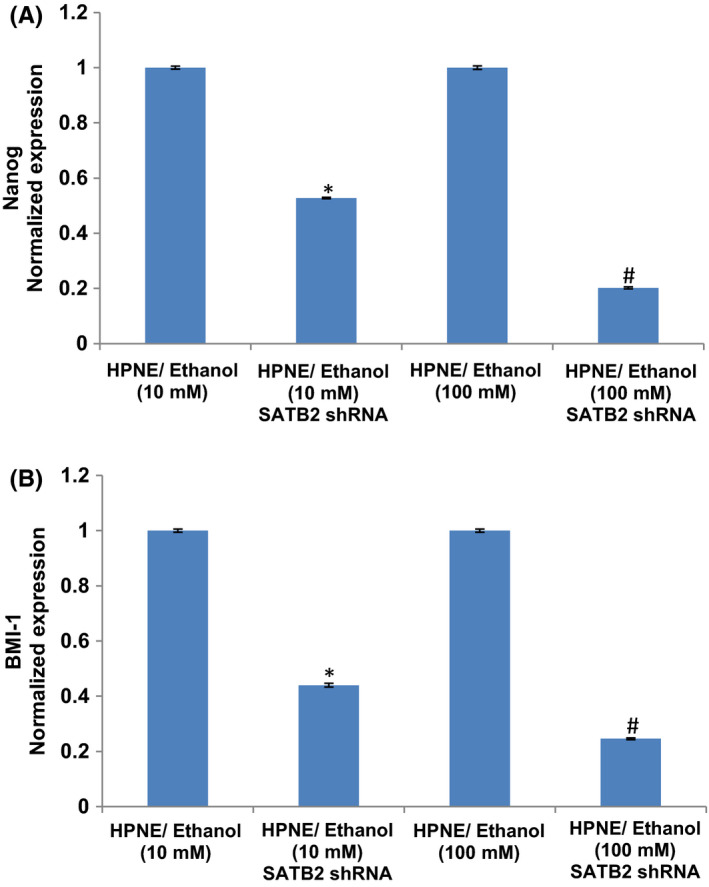
SATB2 shRNA inhibits EtOH‐induced expression of Nanog and BMI in EtOH‐transformed cells. (A–B) Ethanol‐transformed HPNE cells [HPNE/ethanol (10 mM)/SATB2 shRNA, and HPNE/ethanol (100 mM)/SATB2 shRNA] were transduced with lentiviral particles expressing either scrambled or SATB2 shRNA. RNA was isolated, and the expression of Nanog and BMI‐1 was measured by qRT‐PCR. Data represent mean ± SD. * = significantly different from scrambled control group, *p* < 0.05

Since ethanol‐transformed HPNE cells demonstrated stemness by overexpressing Nanog and BMI‐1, we next sought to examine whether inhibition of SATB2 will attenuate the expression of these genes in ethanol‐transformed cells (Figure 6A,B). SATB2 shRNA inhibited the expression of Nanog and BMI‐1 in ethanol‐transformed HPNE cells [HPNE/ethanol (10 mM)/SATB2 shRNA, and HPNE/ethanol (100 mM)/SATB2 shRNA]. These data suggest that SATB2 can regulate stemness by regulating the expression of Nanog and BMI‐1 in ethanol‐transformed HPNE cells (HPNE/ethanol).

### 3.6 Ethanol promotes pancreatic cancer growth and development in KC (Pdx1‐Cre and LSL‐Kras^G12D^) mice

**FIGURE 7:**
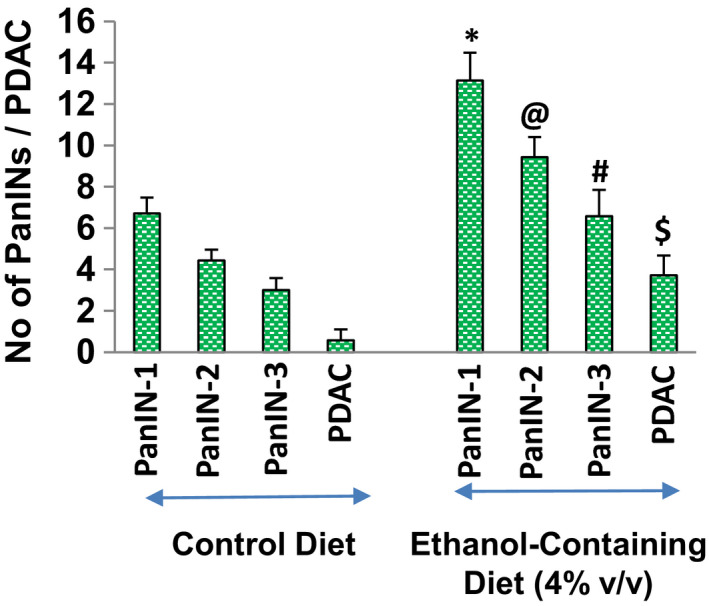
Effects of ethanol feeding on KC (Pdx1‐Cre, and LSL‐Kras^G12D^) mice. KC (Pdx1‐Cre, and LSL‐Kras^G12D^) mice were generated as described elsewhere.^65^ Mice (about 6 weeks old, *n* = 7) were fed with either control diet or ethanol‐containing diet. At the end of the experiment, mice were sacrificed and pancreatic tissues were isolated as described in Materials and Methods. Histological examination of the pancreas was performed by H&E staining. Numbers of PanINs and PDAC were quantified as described elsewhere^36^

Since KC mice mimic pancreatic cancer development in humans, we have used this model to examine whether ethanol promotes pancreatic cancer growth and development in KC (Pdx1‐Cre, and LSL‐Kras^G12D^) mice. KC Mice were fed either a control or ethanol‐containing liquid diet (4%, v/v) for 6 months. KC mice fed with a control diet developed all stages of PanIN lesions (PanIn‐1, PanIN‐2, and PanIn‐3) and PDAC (Figure 7). The development of PDAC was significantly lower than PanINs. Interestingly, mice fed with an ethanol‐containing liquid diet developed significantly higher numbers of PanIN lesions and PDAC than those fed with a control diet. These data suggest that feeding of mice with ethanol can promote pancreatic cancer growth and development in KC mice.

### 3.7 Ethanol feeding enhances stem cell markers, pluripotency‐maintaining factors N‐Cadherin, EMT‐transcription factors and inflammatory cytokines, and inhibits E‐cadherin expression in KC (Pdx1‐Cre and LSL‐Kras^G12D^) mice

**FIGURE 8:**
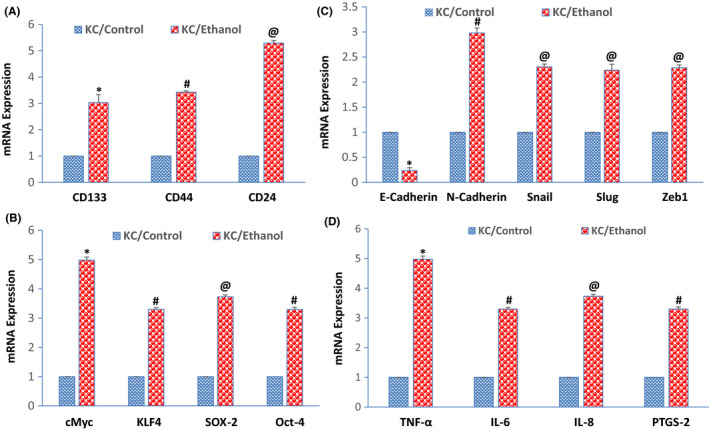
SATB2 shRNA inhibits EtOH‐induced expression of Nanog and BMI in EtOH‐transformed cells. (A) Expression of stem cell markers. RNA was isolated form pancreatic tissues of KC mice fed with an ethanol‐containing diet or a control diet, and the expression of CD133, CD44, and CD24 was measured by qRT‐PCR. Data represent mean ± SD. *, #, or @ = significantly different from scrambled control group, *p* < 0.05. (B) Expression of cMyc, KLF4, SOX‐2, and Oct‐4. RNA was isolated from pancreatic tissues of KC mice fed with either an ethanol‐containing diet or a control diet, and the expression of cMyc, KLF4, SOX‐2, and Oct‐4 was measured by qRT‐PCR. Data represent mean ± SD. *, #, or @ = significantly different from scrambled control group, *p* < 0.05. (C) Expression of cadherins and EMT‐related transcription factors. RNA was isolated from pancreatic tissues of KC mice fed with either an ethanol‐containing diet or a control diet, and the expression of E‐cadherin, N‐cadherin, Snail, Slug, and Zeb1 was measured by qRT‐PCR. Data represent mean ± SD. *, #, or @ = significantly different from scrambled control group, *p* < 0.05. (D) Expression of cytokines and PTGS‐2. RNA was isolated form pancreatic tissues of KC mice fed with either an ethanol‐containing diet or a control diet, and the expression of TNF‐α, IL‐6, IL‐8, and PTGS‐2 was measured by qRT‐PCR. Data represent mean ± SD. *, #, or @ = significantly different from scrambled control group, *p* < 0.05

Since ethanol feeding of KC mice promotes pancreatic cancer growth and development, we next sought to measure the expression of stem cell markers, pluripotency‐maintaining factors cadherins, EMT‐transcription factors and inflammatory cytokines, and PTGS‐2 gene in KC mice. Mice fed with an ethanol‐containing diet showed higher expression of stem cell markers (CD133, CD44, and CD24), and pluripotency‐maintaining factors (cMyc, KLF4, SOX‐2, and Oct‐4) in the pancreas than those fed with a control diet (Figure 8A,B). We next examined whether ethanol feeding regulates EMT‐related genes. KC mice fed with an ethanol‐containing diet showed higher expression of N‐cadherin and EMT‐related transcription factors (Snail, Slug, and Zeb1), and inhibited E‐cadherin in the pancreas than those fed with a control diet (Figure 8C).

In mice, mutant Kras causes spontaneous infiltration of immune cells, which initiate the development of pancreatic cancer.^45^ Inflammatory cytokines and infiltrated immune cells are essential for the initiation and progression of pancreatic cancer.^45^, ^46^, ^47^ We next examined whether ethanol feeding regulates inflammatory cytokines (TNF‐α, IL‐6, and IL‐8) and PTGS‐2 gene (encodes for COX‐2) in the pancreas of KC mice. Mice fed with an ethanol‐containing diet showed higher expression of TNF‐α, IL‐6, IL‐8, and PTGS‐2 gene than those fed with control diet (Figure 8D). Overall, these data suggest that ethanol feeding of KC mice can promote pancreatic cancer growth and development by enhancing stemness and creating inflammatory environments in the pancreas.

## 4 DISCUSSION

The present study demonstrates the carcinogenic effects of alcohol on pancreatic cancer. We have recently shown that during ethanol‐induced malignant transformation, CSCs/ progenitor cells are developed, which may play a significant role in pancreatic carcinogenesis.^23^ We have extended our previous observations and here showed that chronic ethanol exposure of HPNE cells enhanced cell motility which is a characteristic of EMT. Chronic ethanol treatment of HPNE cells induced SATB2, inhibited E‐cadherin, and upregulated N‐cadherin and transcription factor Snail, Slug, Zeb1, Nanog, and BMI‐1. Inhibition of SATB2 expression by shRNA attenuated the effects of ethanol on cell motility and expression of E‐Cadherin, N‐Cadherin, Snail, Slug, and Zeb1. Furthermore, SATB2 shRNA inhibited Nanog and BMI‐1 expression in ethanol‐treated HPNE cells. KC mice fed with an ethanol‐containing diet demonstrated enhanced pancreatic cancer growth and development.

Furthermore, pancreas derived from KC mice fed with an ethanol‐containing diet expressed higher levels of pluripotency, and self‐renewal genes (Oct‐4, SOX‐2, cMyc, and KLF4), stem cell markers (CD24, CD44, and CD133), and N‐cadherin, and lower expression of E‐Cadherin than those derived from control mice. Our data strongly support the idea that ethanol promotes pancreatic cancer growth and development in KC mice by generating CSCs. The conversion of HPNE cells to cancer stem‐like cells by ethanol confirms alcohol as a risk factor for pancreatic cancer.

Epidemiological data suggest that heavy alcohol drinking increases the risk for pancreatic cancer.^6^, ^10^, ^48^, ^49^ Genetic and other environmental factors may further potentiate the adverse effects of alcohol. In mice, while mutant Kras itself causes spontaneous infiltration of immune cells, the additional chronic inflammatory damage further enhances the progression of pancreatic cancer.^45^ Infiltrated immune cells in pancreatic cancer are essential for the initiation and progression of pancreatic cancer, and produce immune‐suppressive signals to dampen antitumour T‐cell responses in tumour.^46^, ^47^ In the present study, we have demonstrated that ethanol feeding can promote pancreatic cancer growth and development in KC mice expressing K‐ras^G12D^ in the pancreas. Ethanol feeding enhanced the production of inflammatory cytokines and PTGS‐2 gene (COX‐2) in KC mice, suggesting an important role of inflammation in carcinogenesis. Similar to our findings, in another study, chronic alcohol intake has been shown to promote intestinal tumourigenesis and tumour invasion in genetically susceptible mice, increase polyp‐associated mast cells, and mast cell‐mediated tumour migration in vitro,^15^ suggesting mast cell‐mediated inflammation could promote carcinogenesis.

We have described the oncogenic role of SATB2 in various cancers, including pancreatic cancer.^23^, ^28^, ^29^, ^30^, ^31^, ^39^ Induction of the SATB2 gene by ethanol was observed in vitro and in vivo. Our previous and current studies demonstrate that ethanol toxicity induces HPNE cell transformation and may induce or enhance, alone or with other factors, pancreatic carcinogenesis by modulating the expression of SATB2. The upregulation of SATB2 in HPNE cells was sufficient to induce malignant transformation in vitro, and those transformed cells gained the phenotypes of CSCs by expressing cancer stem cell markers and pluripotency‐maintaining factors.^28^, ^30^ Furthermore, SATB2‐transformed cells demonstrated high cell cycling and proliferative capabilities, which were associated with Cyclin D1 and Bcl‐2 expression.^28^, ^30^ SATB2 is highly expressed in CSCs isolated from various solid tumours.^28^, ^30^ Although the expression of SATB2 was absent or very low in human pancreatic normal ductal epithelial cells, mammary epithelial cells, and colorectal epithelial cells,^28^, ^29^, ^30^ overexpression of SATB2 in these normal cells induced malignant transformation, suggesting an oncogenic role of SATB2 in cancer. SATB2 alone was capable of regulating pluripotency and self‐renewal because SATB2 binding sites are found in the promoter regions of KLF4, Oct‐4, cMyc, and SOX‐2.^50^, ^51^, ^52^, ^53^

Epithelial–mesenchymal transition plays a significant role in metastasis. Nanog regulates stemness by determining the self‐renewal capacity of embryonic stem cell, and reprogramming of differentiated somatic cells to pluripotency. Nanog is a member of ANTP class NK family genes and plays a key role in stem cell self‐renewal and pluripotency differentiation.^54^ Furthermore, the abnormal expression of the Nanog gene has been reported in malignant germ cell tumours and solid tumours.^28^, ^30^, ^33^, ^55^, ^56^ In the present study, in addition to SNAIL, Slug, and Zeb1, ethanol also induced Nanog in vitro and in vivo. The induction of Nanog and BMI‐1 by ethanol may play a role in EMT and metastasis.

B‐lymphoma Moloney murine leukaemia virus insertion region‐1 (BMI‐1) belongs to a member of the PcG family of transcription repressors.^57^, ^58^ BMI‐1 is involved in carcinogenesis and metastasis.^59^ Upregulation of BMI‐1 was associated with the invasion of nasopharyngeal carcinomas and predicted poor survival.^60^ In the present study, we have shown that chronic ethanol exposure of HPNE cells induces BMI‐1 in vitro, and KC mice fed with ethanol‐containing diet expressed higher level of BMI‐1 in the pancreas than those in control mice. In other studies, BMI‐1 promoted invasion and metastasis of pancreatic cancer stem cells.^44^, ^59^, ^61^, ^62^, ^63^ In another study, BMI‐1 enhanced the invasion and migration of CCSCs through the downregulation of E‐cadherin, possibly by inducing EMT.^62^

Alcohol is a risk factor for pancreatic cancer. KC mice harbour oncogenic Kras mutations in the pancreas and are thus believed to mimic pancreatic cancer development in humans. We have observed all the stages of pancreatic cancer in KC mice fed with a control diet. When KC mice were fed with an ethanol‐containing liquid diet, pancreatic cancer growth and development were accelerated which was evidenced by higher numbers of PanIN lesions and PDAC. Pancreas isolated from KC mice fed with an ethanol‐containing diet also showed higher expression of SATB2, pluripotency, and self‐renewal genes (Oct‐4, SOX‐2, cMyc, and KLF4), stem cell markers (CD24, CD44, and CD133), and N‐cadherin than those from mice fed with a control diet, suggesting that ethanol is capable of promoting stem cell characteristics in KC mice. KC mice fed with an ethanol‐containing diet also showed higher expression of inflammatory cytokines (TNF‐α, IL‐6, and IL‐8) and PTGS‐2 than those fed with a control diet, suggesting an essential role of inflammation in promoting the effects of ethanol on pancreatic carcinogenesis in KC mice. Similarly, a recent finding has reported that moderate alcohol intake promoted pancreatic ductal adenocarcinoma in *Pdx1* ^Cre^;LSL‐*Kras* ^G12D^ mice but not in the control *Pdx1* ^Cre^ mice.^64^ We and others have not observed any induction of pancreatic cancer by alcohol without oncogenic Kras^G12D^ mutations.^64^ The reasons for differences observed in gene expressions of pancreatic ductal epithelial cells by chronic ethanol exposure in vitro compared with ethanol feeding of mice are not known. However, it may be due to the fact that mice are capable of detoxifying adverse effects of alcohol which will be absent in vitro studies.

In conclusion, our data demonstrate that chronic ethanol exposure of HPNE cells induces EMT in vitro, and oral ethanol feeding of KC mice promoted pancreatic cancer growth and development by regulating SATB2, inflammatory cytokines, PTGS‐2, stem cell markers, and pluripotency‐maintaining factors. These data suggest that alcohol is capable of promoting carcinogenesis and metastasis and could harm human health.

## CONFLICT OF INTEREST

All the authors have declared that no conflict of interest exists.

## AUTHOR CONTRIBUTIONS

**Wei Yu:** Conceptualization (equal); Data curation (equal); Formal analysis (equal); Methodology (equal); Project administration (equal); Writing – original draft (equal). **Yiming Ma:** Conceptualization (equal); Data curation (equal); Formal analysis (equal); Methodology (equal); Writing – original draft (equal). **Sanjit K Roy:** Conceptualization (equal); Data curation (equal); Formal analysis (equal); Methodology (equal); Project administration (equal); Writing – original draft (equal). **Rashmi Srivastava:** Writing – original draft (equal). **Sharmila Shankar:** Project administration (equal); Resources (equal); Supervision (equal). **Rakesh K. Srivastava:** Supervision (equal); Writing – review & editing (equal).
